# Comparison of atherogenic indices for predicting the risk of metabolic syndrome in Southwest Iran: results from the Hoveyzeh Cohort Study (HCS)

**DOI:** 10.1186/s13098-024-01349-1

**Published:** 2024-05-23

**Authors:** Hossein Babaahmadi‐Rezaei, Maedeh Raeisizadeh, Vahid Zarezade, Kourosh Noemani, Ahmad Mashkournia, Hossein Ghaderi-Zefrehi

**Affiliations:** 1 Hyperlipidemia Research Center, Department of Clinical Biochemistry, Faculty of Medicine, Ahvaz Jundishapur University of Medical Sciences, Ahvaz, Iran; 2 Department of Biostatistics and Epidemiology, School of Public Health, Ahvaz Jundishapur University of Medical Sciences, Ahvaz, Iran; 3 Behbahan Faculty of Medical Sciences, Behbahan, Iran; 4 Department of Disease Prevention and Control, Deputy of Health Center, Ahvaz Jundishapur University of Medical Sciences, Ahvaz, Iran; 5 Department of Internal Medicine, Ahvaz Jundishapur University of Medical Sciences, Ahvaz, Iran

**Keywords:** Metabolic syndrome, Atherogenic indices, Insulin resistance, Lipids, Cohort

## Abstract

**Background:**

Metabolic syndrome (MetS) is a cluster of risk factors related to diabetes and cardiovascular disease (CVD). Given that early identification of MetS might decrease CVD risk, it is imperative to establish a simple and cost-effective method to identify individuals at risk of MetS. The purpose of this study was to explore the relationships between several atherogenic indices (including AIP, TyG index, non-HDL-C, LDL-c/HDL-c, and TC/HDL-c) and MetS, and to assess the ability of these indices to predict MetS.

**Methods:**

The present cross-sectional study was conducted using baseline data from 9809 participants of the Hoveyzeh Cohort Study (HCS). MetS was defined based on the International Diabetes Federation (IDF). To examine the discriminatory abilities of each atherogenic indices in the identification of MetS, a receiver-operating characteristic curve was conducted. Logistic regression analysis was also performed to evaluate the relationship between atherogenic indices and MetS.

**Results:**

All of the atherogenic indices including the TyG index, AIP, non-HDL-C, TC/HDL-c, and LDL-c/HDL-c were significantly higher in participants with MetS than in those without MetS. According to the ROC curve analysis, the TyG index revealed the highest area under the curve (0.79 and 0.85 in men and women, respectively), followed by the AIP (0.76 and 0.83 in men and women, respectively). The best cutoff values for the TyG index and AIP were 8.96 and 0.16 for men and 8.84 and 0.05 for women, respectively. The TyG index and AIP were also strongly associated with MetS.

**Conclusion:**

Among the 5 atherogenic indices evaluated, the TyG index and AIP were strongly related to MetS. The TyG index also demonstrated superior discriminative ability compared to other atherogenic indices in predicting MetS.

## Introduction

A range of metabolic abnormalities, such as hypertension, abdominal obesity, disrupted glucose metabolism, and dyslipidemia, collectively define the hallmarks of metabolic syndrome (MetS) [1]. The prevalence rate of MetS is estimated to be more than 30% among Iranian adults [2]. A substantial body of evidence links obesity and insulin resistance (IR) to the pathophysiology of MetS. IR, characterized by the inability of tissues to respond adequately to insulin for the regulation of blood glucose, has emerged as a pivotal component in the development of MetS [3–5]. Cardiovascular disease (CVD), representing the most life-threatening risk associated with MetS, is tightly linked to disturbances in lipid metabolism, notably establishing atherosclerosis [6, 7]. Despite advances in techniques and prevention, patients with CVD are still prone to recurrent adverse events at higher rates. Identifying individuals at an early stage who are at a higher risk for CVD will have a notable impact on improving risk stratification and therapeutic management, demonstrating its clinical significance.

Based on the Clinical Practice Guidelines, it is crucial to reduce the plasma concentrations of low-density lipoprotein cholesterol (LDL-c) in patients with Mets [8]. Despite having normal plasma LDL-c levels, patients with MetS and dyslipidemia still face a significant risk of cardiovascular and metabolic events [9]. Therefore, simply concentrating on the LDL-C level is insufficient. The focal point of recent research has been on investigating the efficacy of new atherogenic indices such as Castelli risk index-I or CRI-I [total cholesterol/high-density lipoprotein cholesterol (TC/HDL-c)], CRI-II (LDL-c/HDL-c), non-HDL-c (TC − HDL-c), TyG index [ln (triglyceride (TG) × fasting blood glucose (FBG)/2)], and atherogenic index of plasma [AIP = log (TG/HDL-c)] in predicting the risk of MetS [9, 10]. A study by Zhang et al. compared the predictive power of atherogenic indices as potential markers for predicting MetS in the Chinese population. They suggested that AIP was a better index to identify MetS than the other lipid parameters [10]. Studies have also demonstrated that there is an association between the TyG index and hypertension, and diabetes mellitus [11, 12]. Furthermore, the excellent predictive value of the TyG index to identify MetS was found in different races and ethnic groups [13–17]. The TyG index has been proposed as an efficient and low-cost marker of IR [18]. However, no study has investigated the association between atherogenic indices and MetS in the Arabic population residing in Iran. Therefore, the purpose of this study was to compare the discriminatory capacity of atherogenic indices including LDL-c/HDL-c, TC/HDL-c, non-HDL-C, TyG index, and AIP in identifying individuals with MetS, both among men and women, and establish the respective cutoff values.

## Method

### Study population

The present study was approved by the Ethics Committee of Ahvaz Jundishapur University of Medical Sciences (Ethical code: IR.AJUMS.REC.1402.079). Written informed consent was obtained from all study participants.

The current cross-sectional study was carried out on 9809 individuals from the baseline data of the Hoveyzeh Cohort Study (HCS). The HCS is a population-based study that has been carried out in Hoveyzeh County, Khuzestan Province, southwest of Iran. It is also a subset of the Prospective Epidemiological Research Studies (PERSIAN) Cohort in Iran [19]. Baseline data from the Hoveyzeh Cohort Study were collected from 2016 to 2018, and 10,009 participants aged 35–70 years old, who were registered as permanent inhabitants of Hoveyzeh, participated in this cohort. Details of the HCS have been published before [20].

### Inclusion and exclusion criteria

**Fig. 1:**
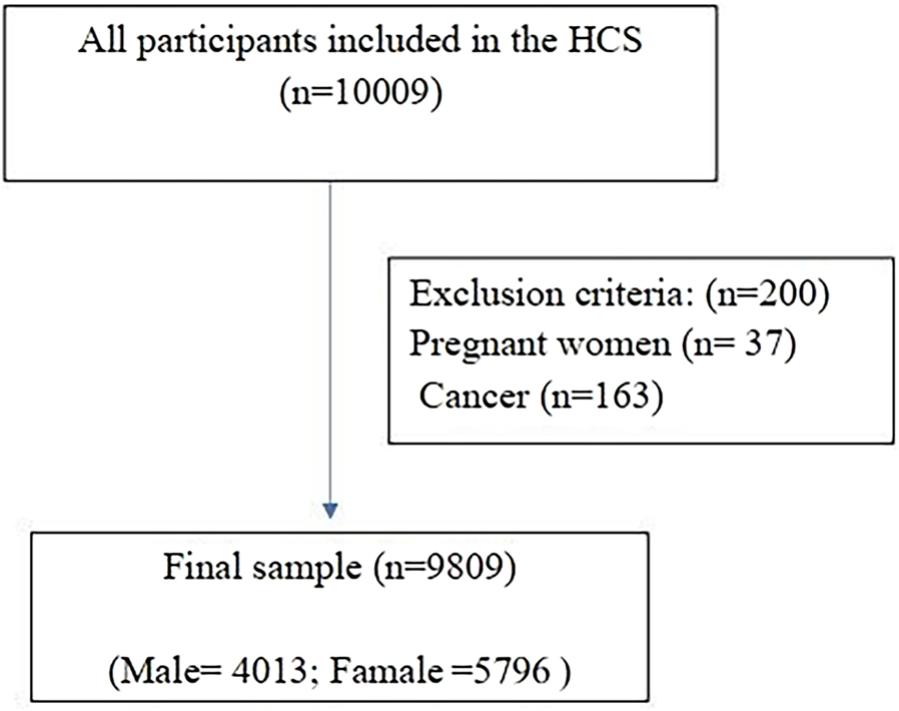
Selection process of the participants

All individuals who participated in the baseline phase of HCS (n = 10,009) entered the current study. Participants with cancer (n = 37) and pregnant women (n = 163) were excluded from the study. Eventually, the final study population was 9809 subjects (4013 men and 5796 women) (Fig. 1).

### Data collection

The demographic data (age, sex, residence type, and level of education), personal habits (smoking status and alcohol consumption), and medication use were gathered through a questionnaire and direct interview. A subject was defined as a smoker if he or she reported smoking at least 100 cigarettes during his or her lifetime [20]. All participants were asked whether they have ever used alcohol (yes or no). The self-report questionnaire was used to assess physical activity based on the metabolic equivalent of task (MET). The degree of physical activity was grouped into four quartiles (sedentary, light, moderate, and vigorous). Anthropometric parameters, diastolic blood pressure (DBP), systolic blood pressure (SBP), and biochemical parameters TG, FBG, HDL-c, and TC were measured according to the PERSIAN cohort protocol.

### Anthropometric parameters and blood pressure measurements

After overnight fasting, anthropometric parameters were measured using an appropriate technique. The weight (kg) and height (cm) of the subjects were measured in light clothing without shoes using a standing scale (Seca 755) and a stadiometer (Seca 206), respectively, and waist circumference was also measured using Seca locked tape meters. BMI (kg/m^2^) was calculated by dividing weight (kg) by the square of the height (m^2^). DBP and SBP were measured in the seated position using Riester sphygmomanometers, twice with 10-min intervals between the two measurements, and the mean was reported.

### Biochemical measurements

After 10–12 h of fasting, blood samples were taken by the trained laboratory staff based on the standard protocol. The blood samples were centrifuged and the serum was separated. Lipid profiles (TG, HDL-c, and TC) and FBG were determined by the enzymatic method by the autoanalyzer (BT 1500, Biotecnica Instruments, Italy). LDL-c was calculated by the Friedewald equation.

### Definition of metabolic syndrome

MetS was defined using the International Diabetes Federation (IDF) criteria. Based on the IDF criteria, waist circumference (WC) ≥ 80 cm in women and ≥ 94 cm in men plus at least two of the following four criteria is defined as MetS: fasting blood sugar ≥ 100 mg/dL, HDL-c level < 50 mg/dL (women) or < 40 mg/dL (men), TG level ≥ 150 mg/dL and BP ≥ 130/85 mmHg [21].

### Atherogenic indices

The atherogenic lipid indices were calculated as follows:

$${\text{TyG}}\;{\text{index}} = {\text{Ln}}\;\left( {{\text{TG}} \times {\text{FBG}}/2} \right),$$

$${\text{Atherogenic}}\;{\text{index}}\;{\text{of}}\;{\text{plasma}}\;\left( {{\text{AIP}}} \right) = \log \;\left( {{\text{TG}}/{\text{HDL-c}}} \right),$$

$${\text{Castelli}}\;{\text{Index}}\;{\text{I}}\;({\text{CRI}}.{\text{I}}) = {\text{TC}}/{\text{HDL-c}},$$

$${\text{Castelli}}\;{\text{Index}}\;{\text{II}}\;\left( {{\text{CRI}}.{\text{II}}} \right) = {\text{LDL-c}}/{\text{HDL-c}},$$

$${\text{Non-HDL-c}} = {\text{TC}} - {\text{HDL-c}}.$$

In these formulas, total TG, HDL-c, LDL-c, and TC were expressed as mmol/L [9].

### Statistical analysis

Outcome measures are presented as the mean ± standard deviation (M ± SD). Student’s t-test and the chi-square test were applied for the comparison of continuous and categorical variables, respectively. Subjects were divided into 4 quartiles according to the values of atherogenic indices. To evaluate the possible association between these atherogenic indices and MetS, odds ratios (ORs) with 95% confidence intervals (CIs) were reported by logistic regression analysis using crude and adjusted models (adjusted for age, BMI, physical activity, and smoking). The area under the curve (AUC) of the receiver operating characteristic (ROC) curve was measured to compare the predictive capacity of the various atherogenic indices for identifying MetS. The best cutoff points were defined by the maximum value of Youden’s index (sensitivity + specificity 1). Data analysis was carried out using SPSS version 22 and medcalc version 2.0. A p-value < 0.05 was considered statistically significant.

## Results

### Baseline characteristics of the participants

The study sample consisted of 9809 participants, of whom 4013 (40.9%) were male. At baseline, 1508 (37.6%) males and 2977 (51.4%) females were diagnosed with MetS. Table 1 presents the baseline characteristics of the subjects with or without MetS. In both sexes, the participants with MetS presented significantly higher weight, WC, DBP, SBP, BMI, FBG, TG, and TC, and lower HDL-c than the participants without MetS (P < 0.001). Height and LDL were only significantly higher in males with MetS than in those without MetS. Furthermore, as demonstrated in Table 1, all of the atherogenic indices including the TyG index, AIP, LDL-c/HDL-c, TC/HDL-c, and non-HDL-C were significantly higher in participants with MetS of both genders.

**Table 1:** Basic characteristics of the study population according to gender and MetS

Parameters	Male (n = 4013)			Female (n = 5796)		
	With MetS	Without MetS	P value	With MetS	Without MetS	P value
N (%)	1508 (37.6%)	2505 (62.4%)		2977 (51.4%)	2819 (48.6%)	
Age (years)	49.93 ± 9.11	48.55 ± 9.24	< 0.001	50.78 ± 9.24	46.27 ± 8.50	< 0.001
Anthropometry						
Height (cm)	173.67 ± 6.23	72.56 ± 6.38	< 0.001	158.93 ± 5.69	158.90 ± 5.64	0.93
Weight (kg)	91.28 ± 12.87	77.15 ± 14.25	< 0.001	77.95 ± 14.26	72.08 ± 15.20	< 0.001
BMI (kg/m^2^)	30.23 ± 3.78	25.86 ± 4.30	< 0.001	30.82 ± 5.22	28.50 ± 5.63	< 0.001
WC (cm)	104.79 ± 8.16	92.59 ± 11.02	< 0.001	104.73 ± 10.8	97.7 ± 12.39	< 0.001
DBP (mmHg)	76.84 ± 11.81	71.36 ± 10.36	< 0.001	72.11 ± 11.38	67.94 ± 10.04	< 0.001
SBP (mmHg)	122.35 ± 18.80	120.45 ± 15.18	< 0.001	115.51 ± 19.94	106.07 ± 15.42	< 0.001
Educational level			0.316			< 0.001
Illiterate	575 (38.1%)	1045 (41.7%)		2363 (79.3%)	2088 (74%)	
Primary school	333 (22%)	504 (20.1%)		386 (12.9%)	404 (14.3%)	
Middle school	187 (12.4%)	278 (11%)		83 (2.7%)	116 (4.1%)	
High school	213 (14.12%)	327 (13%)		85 (2.8%)	106 (3.7%)	
University	200 (13.26%)	351 (14%)		60 (2%)	105 (3.7%)	
Residence type			< 0.001			< 0.001
Urban	1010 (66.9%)	1513 (60.3%)		1991 (66.8%)	1541 (54.6%)	
Rural	498 (33%)	992 (39.6%)		986 (33.1%)	1278 (45.3%)	
Smoker	610 (40.4%)	1019 (40.6%)	0.457	266 (8.9%)	173 (6.1%)	< 0.001
Use of alcohol (yes)	69 (4.5%)	120 (4.7%)	0.410	5 (0.1%)	1 (0.0%)	0.123
Physical activity			< 0.001			< 0.001
Q_1_	533 (35.3%)	716 (28.5%)		781 (26.2%)	425 (15%)	
Q_2_	295 (19.5%)	436 (17.4%)		881 (29.5%)	830 (29.4%)	
Q_3_	241 (15.9%)	407 (16.2%)		832 (27.9%)	969 (34.3%)	
Q_4_	439 (29.1%)	946 (37.7%)		483 (16.2%)	595 (21.1%)	
Medication use						
Lipid-lowering drugs	16 (1%)	15 (0.5%)	0.135	30 (1%)	4 (0.1%)	< 0.001
Glucose-lowering drugs	161 (10.6%)	209 (8.3%)	0.015	318 (10.6%)	168 (5.9%)	< 0.001
Antihypertensive drugs	214 (14.1%)	206 (8.2%)	< 0.001	514 (17.2%)	123 (4.3%)	< 0.001
Dietary						
Energy (kcal/day)	3440.11 ± 1050.85	3373.98 ± 1013.01	0.048	2618.10 ± 790.09	2786.28 ± 847.51	< 0.001
Protein (g/day)	114.10 ± 35.46	110.09 ± 33.44	< 0.001	85.22 ± 25.77	88.85 ± 27.29	< 0.001
Fat (g/day)	67.93 ± 28.56	68.44 ± 27.28	0.573	55.10 ± 22.82	60.68 ± 25.74	< 0.001
Carbohydrate (g/day)	602.91 ± 188.59	589.28 ± 187.24	0.026	454.80 ± 144.11	480.78 ± 152.94	< 0.001
Laboratory data						
FBG (mg/dL)	129.84 ± 58.25	103.57 ± 43.39	< 0.001	128.34 ± 60.07	95.51 ± 27.30	< 0.001
TG (mg/dL)	235.55 ± 149.37	151.39 ± 95.39	< 0.001	189.92 ± 110.09	109.81 ± 45.03	< 0.001
TC (mg/dL)	189.35 ± 41.90	185.30 ± 37.82	0.005	195.08 ± 43.37	186.70 ± 37.61	< 0.001
HDL-c (mg/dL)	42.43 ± 9.58	48.14 ± 10.23	< 0.001	48.95 ± 11.39	57.29 ± 11.50	< 0.001
LDL-c (mg/dL)	101.11 ± 34.05	107.3 ± 30.84	< 0.001	108.44 ± 34.85	107.50 ± 31.71	0.183
Atherogenic indices						
AIP	0.33 ± 0.25	0.09 ± 0.25	< 0.001	0.19 ± 0.23	− 0.09 ± 0.19	< 0.001
TC/HDL-c	4.59 ± 1.14	3.95 ± 0.92	< 0.001	4.10 ± 0.97	3.33 ± 0.71	< 0.001
LDL-c/HDL-c	2.41 ± 0.77	2.28 ± 0.69	< 0.001	2.27 ± 0.73	1.93 ± 0.62	< 0.001
Non-HDL-c (mmol/L)	3.79 ± 1	3.54 ± 0.92	< 0.001	3.77 ± 1.03	3.34 ± 0.9	< 0.001
TyG index	9.42 ± 0.61	8.79 ± 0.57	< 0.001	9.22 ± 0.61	8.47 ± 0.42	< 0.001

Data are presented as mean ± standard deviation or n (percent) MetS, metabolic syndrome

*BMI* body mass index, *WC* waist circumference, *DBP* diastolic blood pressure, *SBP* systolic blood pressure, *FBG* fasting blood glucose, *TG* triglycerides, *TC* total cholesterol, *HDL-C* high-density lipoprotein-cholesterol, *LDL-C* low-density lipoprotein-cholesterol, *AIP* atherogenic index of plasma, *TyG* triglyceride-glucose

### ROC curves and AUC for atherogenic indices in identifying MetS

**Fig. 2:**
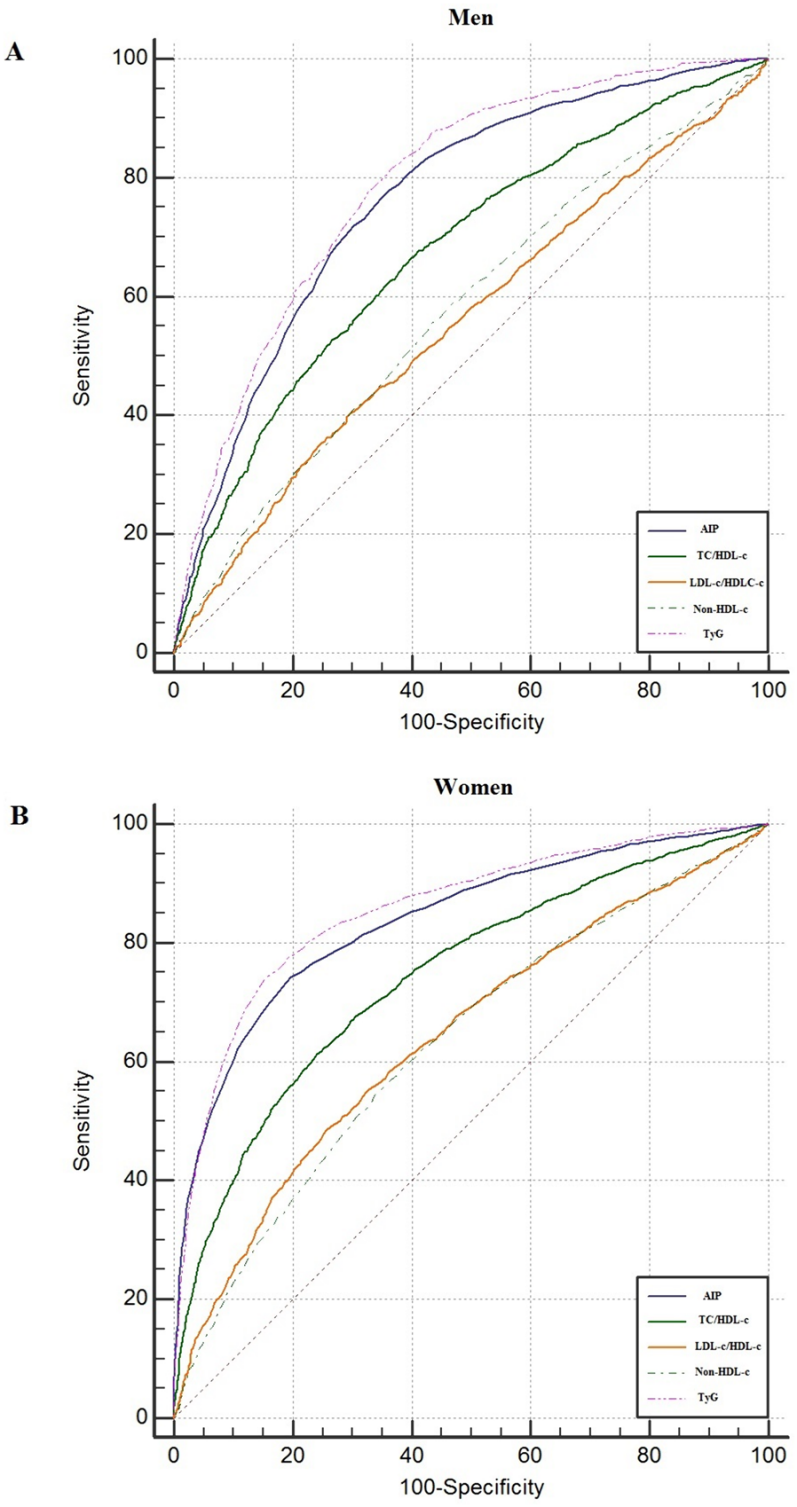
ROC curves for the five atherogenic indices to predict MetS in men (**A**) and women (**B**)

AUC values [95% CI] of the atherogenic indices for identifying subjects with MetS are demonstrated in Table 2 and Fig. 2. All five atherogenic indices evaluated in the present study were able to discriminate MetS [(AUC) > 0.6, P < 0.05] in both men and women, except for LDL-c/HDL-c and non-HDL-C in men. Of the five indices investigated, the TyG index had the highest AUC value in both women (0.85, 95% CI 0.84–0.86) and men (0.79, 95% CI 0.77–0.80), followed by AIP in both women (0.83, 95% CI 0.82–0.84) and men (0.76, 95% CI 0.75–0.77). The best cutoff values of the TyG index to identify MetS were 8.84 (sensitivity 74.5, specificity 84.2) in women and 8.96 (sensitivity 77.9, specificity 67.1) in men. The best cutoff values for AIP were 0.16 in men and 0.05 in women.

**Table 2:** The AUCs, optimal cut-off values, sensitivity, specificity, and Youden index of the five atherogenic indices for predicting MetS

Parameters	AUC	95% CI	Cut-off	Sen	Spe	Youden’s Index	P value
Male							
AIP	0.764	0.751 to 0.777	0.16	76.79	65.03	0.41	< 0.001
TC/HDL-c	0.676	0.661 to 0.690	4.13	65.32	61.36	0.26	< 0.001
LDL-c/HDL-c	0.556	0.540 to 0.572	2.6	39.89	70.91	0.1	< 0.001
Non-HDL-c	0.577	0.561 to 0.592	3.62	55.7	56.7	0.12	< 0.001
TyG	0.789	0.776 to 0.801	8.96	77.98	67.19	0.45	< 0.001
Female							
AIP	0.837	0.827 to 0.847	0.05	74.27	80.36	0.54	< 0.001
TC/HDL-c	0.743	0.732 to 0.754	3.77	61.34	75.88	0.37	< 0.001
LDL-c/HDL-c	0.643	0.630 to 0.655	2.27	48.52	74.33	0.22	< 0.001
Non-HDL-c	0.632	0.619 to 0.644	3.59	54.4	66.2	0.2	< 0.001
TyG	0.855	0.846 to 0.864	8.84	74.5	84.2	0.58	< 0.001

*AIP* atherogenic index of plasma, *TC* total cholesterol, *HDL-c* high-density lipoprotein cholesterol, *LDL-c* low-density lipoprotein cholesterol, *TyG index* triglyceride-glucose, *CI* confidence interval, *sen* sensitivity, *spe* specificity, *AUC* area under the curve

### Odds ratios for MetS risk across quartiles of atherogenic indices

Table 3 presents both the unadjusted and the adjusted ORs (95% CIs) of atherogenic indices for MetS in men and women. In the unadjusted logistic regression analysis, all atherogenic indices were significantly positively correlated with the risk of MetS in both men and women, except for LDL-c/HDL-c (second and third quartiles) and non-HDL-C (second quartile) in men. In the adjusted logistic regression analysis, all atherogenic indices were significantly positively associated with the risk of MetS in both men and women, except for AIP (second quartile), TC/HDL-c (second quartile), LDL-c/HDL-c (second and third quartiles), and non-HDL-C (second and third quartiles) in men.

**Table 3:** The MetS risk across quartiles of atherogenic indices

	Male				Female			
	Model 1 OR (95% CI)	P value	Model 2 OR (95% CI)	P value	Model 1 OR (95% CI)	P value	Model 2 OR (95% CI)	P value
AIP								
Q1; < − 0.08	1 (Reference)		1 (Reference		1 (Reference)		1 (Reference	
Q2; − 0.08–0.09	1.83 (1.34–2.50)	< 0.001	1.41 (0.99–1.98)	0.051	2.79 (2.39–3.26)	< 0.001	2.50 (2.12–2.95)	< 0.001
Q3; 0.09–0.27	5.76 (4.34–7.64)	< 0.001	4.78 (3.49–6.56)	< 0.001	12.56 (10.6–14.89)	< 0.001	11.78 (9.84–14.11)	< 0.001
Q4; > 0.27	15.21 (11.54–20.06)	< 0.001	14.63 (10.66–20.07)	< 0.001	77.14 (57.48–103.52)	< 0.001	77.56 (57.17–105.24)	< 0.001
TC/HDL-c								
Q1; < 3.21	1 (Reference)		1 (Reference)		1 (Reference)		1 (Reference)	
Q2; 3.21–3.81	1.36 (1.04–1.74)	0.014	1.26 (0.95–1.67)	0.1	2.03 (1.76–2.34)	< 0.001	1.86 (1.60–2.17)	< 0.001
Q3; 3.81–4.49	2.26 (1.79–2.84)	< 0.001	1.89 (1.46–2.46)	< 0.001	4.64 (3.99–5.40)	< 0.001	4.13 (3.52–4.85)	< 0.001
Q4; > 4.49	4.93 (3.95–6.14)	< 0.001	4.34 (3.37–5.60)	< 0.001	14.27 (11.73–17.37)	< 0.001	12.77 (10.39–15.67)	< 0.001
LDL-c/HDL-c								
Q1; < 1.69	1 (Reference)		1 (Reference)		1 (Reference)		1 (Reference)	
Q2; 1.69–2.15	1.05 (0.86–1. 29)	0.60	0.99 (0.78–1.25)	0.91	1.31 (1.14–1.51)	< 0.001	1.23 (1.06–1.43)	0.006
Q3; 2.15-.2.64	1.04 (0.85–1.27)	0.65	0.91 (0.72–1.15)	0.43	2.28 (1.97–2.64)	< 0.001	2.02 (1.73–2.36)	< 0.001
Q4; > 2.64	1.62 (1.34–1.96)	< 0.001	1.33 (1.07–1.65)	0.01	3.89 (3.32–4.57)	< 0.001	3.28 (2.75–3.86)	< 0.001
Non-HDL-c								
Q1; < 2.92	1 (Reference)		1 (Reference)		1 (Reference)		1 (Reference)	
Q2; 2.92–3.51	1.17 (0.96–1.42)	0.112	0.99 (0.79–1.25)	0.99	1.37 (1.18–1.59)	< 0.001	1.28 (1.09–1.50)	0.002
Q3; 3.51–4.16	1.51 (1.25–1.83)	< 0.001	1.18 (0.95–1.47)	0.13	2.10 (1.81–2.43)	< 0.001	1.77 (1.51–2.07)	< 0.001
Q4; > 4.16	1.98 (1.64–2.38)	< 0.001	1.48 (1.19–1.84)	< 0.001	3.51 (3.01–4.09)	< 0.001	2.64 (2.24–3.11)	< 0.001
TyG								
Q1; < 8.47	1 (Reference)		1 (Reference)		1 (Reference)		1 (Reference)	
Q2; 8.47–8.85	2.99 (2.16–4.14)	< 0.001	2.05 (1.45–2.91)	< 0.001	2.54 (2.14–3)	< 0.001	2.06 (1.73–2.45)	< 0.001
Q3; 8.85–9.30	10.31 (7.59–14.01)	< 0.001	7.54 (5.40–10.51)	< 0.001	15.40 (12.91–18.36)	< 0.001	12.63 (10.54–15.14)	< 0.001
Q4; > 9.30	26.12 (19.23–35.47)	< 0.001	20.74 (14.83–29)	< 0.001	64.73 (50.31–82.86)	< 0.001	52.47 (40.48–68.02)	< 0.001

Model 1: crude model. Model 2: adjusted for age, BMI, physical activity, energy intake, and smoking

*AIP* atherogenic index of plasma, *TC* total cholesterol, *HDL-c* high-density lipoprotein cholesterol, *LDL-c* low-density lipoprotein cholesterol, *TyG index* triglyceride-glucose, *CI* confidence interval, *OR* odds ratio

## Discussion

In this study, we evaluated and compared the predictive power and cutoff points of atherogenic indices in recognizing MetS in the Iranian Arab population. The findings demonstrated that the TyG index had better predictive capacity for MetS in both men and women. Furthermore, logistic regression analysis revealed that atherogenic indices, in particular, AIP and the TyG index, were related to an elevated risk of MetS, and this association was prominent in women.

The high prevalence rate of metabolic syndrome in recent years makes it necessary to establish effective and simple indices that facilitate the identification of MetS in clinical practice [22].

Among the atherogenic indices, the TyG index has received special attention. The TyG index was indicated to be an effective predictor of diabetes, non-alcoholic fatty liver disease, and ischemic stroke [23–25]. Furthermore, the TyG index is related to IR and has been proposed as a useful surrogate for IR, the hallmark of MetS [26]. The TyG index has better specificity and sensitivity than the gold standard method for measuring IR [27]. The TyG index, which is easily calculated from TG and FBG levels, is less expensive and more accessible than insulin-based indices. Compared with TG and FBG, the TyG index has better predictive power for predicting MetS [28]. Various studies have confirmed the predictive ability of the TyG index for MetS [9, 13–15, 29]. Fernández-Aparicio et al. investigated the ability of atherogenic indices to predict MetS in Spanish adolescents and found that the TyG index had the greatest AUC in males and the TyG index and LDL-c/HDL-c had the greatest AUCs in females [9]. Duan et al. demonstrated that the TyG index had a good predictive value (AUC between 0.841 and 0.886) in Chinese adults [14]. Another study in Thailand found that the TyG index had excellent predictive ability for MetS in law enforcement officers (AUC = 0.88) [16]. Furthermore, the excellent predictive value of the TyG index to identify MetS was found in Polish [15], Korean [17], and Taiwanese populations [13]. Our study demonstrated that the TyG index had the highest AUC for MetS prediction in both men and women (males AUC = 0.79, females AUC = 0.85), consistent with many previous studies. In the current study, the best cutoff of the TyG index to identify MetS was 8.96 in males and 8.84 in females. A similar cutoff value was suggested in middle-aged and elderly Chinese individuals (8.9 for males and 8.7 for females) [30] and in American adults (8.82 for males and 8.68 for females) [31]. Since the TyG index incorporates FBG along TG, the superiority of TyG in predicting MetS may be achieved by simultaneously considering FBG and TG, both of which play an important role in IR [32, 33]. TG and FBG primarily reflect IR in adipose tissue and the liver, respectively [34, 35]. Therefore, the TyG index presents a more complete evaluation of IR. Consistent with the findings of Li et al. [31] and Rattanatham et al. [16], we also confirmed an increase in MetS risk across TyG index quartiles. Compared with quartile 1, quartile 4 of TyG index was related to an elevated risk of MetS with an OR of 20.74 (95% CI 14.83–29, P < 0.001) in men and 52.47 (95% CI 40.48–68.02, P < 0.001) in women. Therefore, the TyG index provides promising prospects for the prediction of MetS.

In recent years, various studies have confirmed the predictive power of the AIP, as a marker of plasma atherogenicity, in MetS, atherosclerosis, diabetes, and non-alcoholic fatty liver disease [36–38]. Compared to LDL-c or TG, the AIP is a better predictor for cardiovascular risk [39]. Studies have also demonstrated that subjects with high AIP index levels have a significantly increased MetS risk [40]. Zhang et al. compared the predictive power of AIP, non-HDL-c, LDL-c/HDL-c, TG/HDL-c, and TC/HDL-c in discriminating MetS in the Chinese population and revealed that AIP was a better index to identify MetS than the other lipid parameters [10]. Vega-Cardenas and colleagues found that AIP had a strong predictive ability for MetS (AUC = 0.91), and the optimal cutoff point was 0.44 [41]. Furthermore, a study conducted among various ethnicities in China showed that AIP has a good predictive performance for MetS, and the cutoff values for AIP ranged from − 0.1 to 0/07 [40]. In the present study, AIP had the second highest AUC value for identifying MetS in both males and females (males AUC = 0.76, females AUC = 0.83). The cutoff values of AIP for predicting MetS were 0.16 in men and 0.05 in women. The discrepancy between the studies’ results may be due to differences in participants’ age, ethnicity, and MetS diagnosis criteria. For instance, in our study, IDF criteria were used to define MetS, while American Heart Association (AHA) criteria were used in Vega-Cardenas et al.’s study [41]. The results of logistic regression exhibited a significant association between the AIP and the MetS risk in both sexes. Compared to quartile 1, the odds of MetS were elevated in quartile 4 of AIP among men (OR = 14.63, CI 10.66–20.07) and women (OR = 77.56, CI 57.17–105.24).

Non-HDL-c reflects the amount of cholesterol within all apoB-containing lipoproteins [42] and could be used as a predictor of CVD and diabetes [43]. A recent meta-analysis revealed that non-HDL-c is strongly correlated with enhanced MetS and its components [44]. We also indicated that non-HDL-c is related to MetS, especially in women. The AUCs of non-HDL-c for men and women were 0.57 and 0.63, respectively, which were lower than those of the Chinese [45] and Polish studies [46]. These differences may be due to differences in the ethnicities of the studied populations.

There are some limitations in this research. First, the participants in this research were an Arab community in southwest Iran, and the results may not be generalizable to other racial and ethnic groups. Second, due to the study’s cross-sectional nature, it is impossible to elucidate a causal association between the variables. Therefore, further studies are needed in this field.

## Conclusion

In the current study, we identified a significant relationship between atherogenic indices and MetS in the Iranian population. The results indicated that the TyG index was superior to other indices in predicting MetS. Given that early identification of MetS might decrease the risk of CVD, it is imperative to establish a simple and cost-effective method to identify individuals at risk of MetS. The TyG index is a simple and cost-effective index, and it only requires serum TG and FBG values. Given the simplicity and superiority of TyG index in identifying MetS, we recommend that the TyG index is a cost-effective screening index to identify individuals at risk of MetS in the clinical setting.

## Data Availability

The datasets used and/or analyzed during the current study are available from the corresponding author upon reasonable request.
